# Increased susceptibility to prostate cancer biomarkers in the offspring of male mouse progenitors with lifelong or early life exposure to high-fat diet

**DOI:** 10.1007/s00394-025-03737-3

**Published:** 2025-06-07

**Authors:** Mariana Santos-Pereira, Sara C. Pereira, Bárbara Matos, Margarida Fardilha, Pedro F. Oliveira, Marco G. Alves

**Affiliations:** 1 Institute of Biomedicine, Department of Medical Sciences, University of Aveiro, Aveiro, 3810-193 Portugal; 2 School of Medicine and Biomedical Sciences (ICBAS), University of Porto, Porto, 4050-313 Portugal; 3 Laboratory for Integrative and Translational Research in Population Health (ITR), University of Porto, Porto, 4099-002 Portugal; 4 Department of Chemistry, LAQV-REQUIMTE, Campus Universitario de Santiago, Aveiro, 3810-193 Portugal

**Keywords:** Obesity, Prostate cancer, Transgenerational, Epigenetic

## Abstract

Obesity exacerbates hormonal dysregulation, inflammation, and oxidative stress, factors associated with Prostate Cancer (PCa) development. The (epi)genetic influences of obesity may be transgenerationally transmitted, potentially impacting PCa susceptibility in the offspring of fathers with obesity. Thus, we studied the impact of early-life or lifelong exposure to a high-fat diet (HFD) on PCa biomarkers [Homeobox B13 (HOXB13) and the Androgen Receptor (AR)] and their correlation with obesity-related markers. Furthermore, we focused on the offspring’s PCa biomarkers outcomes and explored their potential link with paternal diet. A transgenerational *Mus musculus* model was established, with F0 males exposed to different diets (200 days): standard chow, lifelong HFD (HFD), and transient diet (60 days HFD, plus 140 days of standard chow) (HFD_t_). *AR* expression in the prostates was unaffected, whereas *HOXB13*, Fat Mass and Obesity Associated gene (*FTO)*, and Tumor Necrosis Factor-Alpha (*TNF-α)* expression decreased in the F1 HFD_t_ group. *HOXB13* and *AR* prostate expression were positively correlated. There was also a positive correlation between *FTO* prostate expression and PCa biomarkers, and between *TNF-α* expression and *FTO* and PCa biomarkers. *HOXB13* promoter methylation levels were unaffected, however, were positively correlated with *FTO* and *HOXB13* expression. Finally, protein nitration remained unchanged in the prostates, while lipid peroxidation was increased in the F0 HFD group and decreased in the F1 and F2 HFD and HFD_t_ groups. Our study highlights the intergenerational interplay between obesity-related factors and PCa biomarkers, suggesting that offspring of male progenitors subjected to HFD may face an increased risk for elevated PCa biomarkers expression.

**Supplementary Information:**

The online version contains supplementary material available at 10.1007/s00394-025-03737-3.

## Introduction

The World Health Organization (WHO) defines obesity as an “abnormal or excessive buildup of fat” and a Body Mass Index (BMI) equal to or exceeding 30 kg/m^2^ [1, 2]. This metabolic disorder has been on the rise, with recent statistics indicating a higher prevalence in the years to come [3, 4]. Obesity is a complex disease influenced by different factors, including genetic predisposition, hormonal dynamics, and environmental cues [5]. It can lead to the development of several comorbidities such as type 2 diabetes, cardiovascular disease, and cancer. In fact, it is estimated that obesity contributes to approximately 20% of all cancer cases [6, 7] and is recognized to promote inflammation, oxidative stress (OS), and hormonal dysregulation. Further, while inflammation promotes OS, OS and adipokines heighten inflammation, perpetuating a deleterious cycle. The interplay between inflammation, OS, and hormonal dysregulation catalyzes carcinogenesis [8]. Conversely, men with obesity often exhibit reduced testosterone levels [9], that are suggested to play a crucial role in cancer development, like Prostate Cancer (PCa) [10]. Men with obesity are at a higher risk of developing PCa, a prevalent malignancy and a leading cause of mortality among men [11]. Traditional PCa diagnostic methods, like the detection of the Prostate-specific Antigen (PSA) and Digital Rectal Examination (DRE) exhibit reduced specificity and increased failure rates in men with obesity. Those men present a larger serum volume, leading to decreased PSA serum concentration and a false negative test, alongside prostate enlargement complicates the DRE and may result in inaccurate diagnosis [12]. Thus, the increased risk for PCa development and a higher probability of a failed diagnosis should be considered in individuals with obesity. The Androgen Receptor (AR) is linked to prostate health and development. Additionally, dysregulation of AR has been linked with PCa progression [13]. In fact, AR has been present in the majority of primary and metastatic PCa and approximately 80 to 90% of the PCa cases depend on androgen at an early stage, hence the first approach tends to be androgen therapy ablation with the goal to decrease androgen levels and suppress AR [14]. Additionally, the complex interaction between the Homeobox B13 (HOXB13) and AR is crucial for prostate growth and differentiation, since HOXB13 regulates AR expression and prostate development. HOXB13 also presents a dual role in PCa development. While HOXB13 suppresses prostate cell proliferation in the PC-3 cell line through the inhibition of androgen-mediated signaling, higher HOXB13 levels are linked to PCa progression [15, 16, 17].

Obesity has been increasingly associated with (epi)genetic mechanisms, which involve the transmission of phenotypic traits across generations, able to influence metabolic pathways [18]. Metabolic disorders, including obesity, were reported to be transgenerationally inherited by the paternal line, affecting up to two subsequent generations [19, 20]. The interplay between genetic predisposition and epigenetic alterations has been implicated in carcinogenesis [21]. PCa appears to have an autosomal dominant inheritance, with accumulating evidence linking it to factors such as excess adiposity [22, 23]. However, the intricate relationship between obesity and PCa transgenerational inheritance remains largely unexplored. We hypothesized that lifelong or early life obesity in male progenitors may influence PCa biomarkers expression in their offspring. Overall, the main objective was to evaluate whether dietary-induced modifications can affect PCa biomarkers and if these effects are potentially transmitted across generations, thereby increasing the PCa risk among descendants.

## Methods

### Animal model

**Fig. 1:**
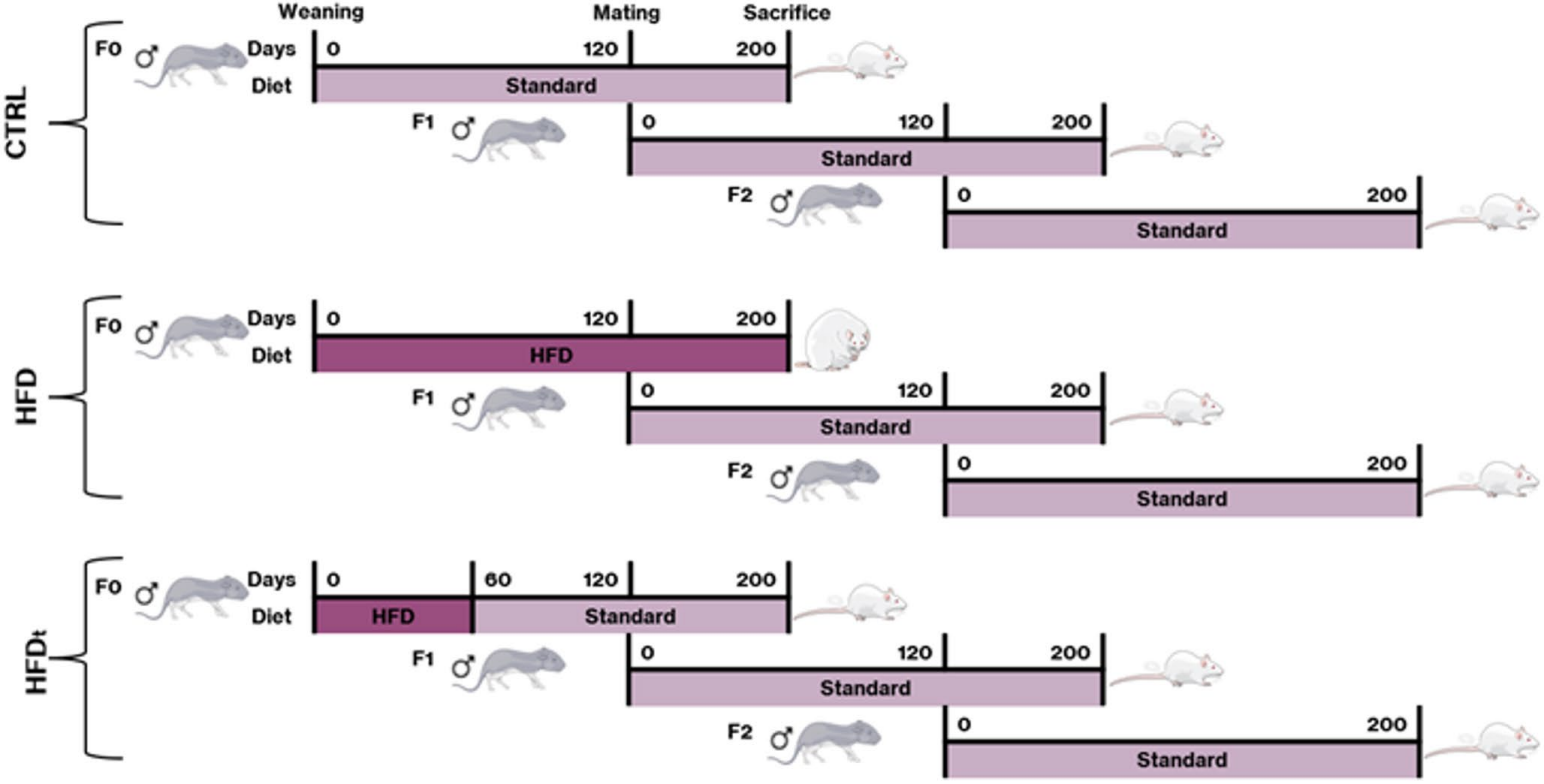
Schematic representation of the transgenerational animal model

For this work, a transgenerational *Mus musculus* C57BL6/J model developed by Crisóstomo L. and colleagues was used [24], with metabolic characterization and serum hormones levels already described [25]. No significant differences were found in F0 or for testosterone levels across all generations. In F1, the HFD_t_ group exhibited decreased levels of 17β-estradiol. Further, in F2, the HFD_t_ group presented higher FSH levels and lower LH levels. In the F2 HFD group, only the levels of 17β-estradiol were augmented [25]. Briefly, normal weight progenitors originated the first generation (F0) fed with standard chow (#F4031, BioServ, USA—Carbohydrate: 61.6%, Protein: 20.5%, Fat: 7.2–16.3% Kcals). Post-weaning (21–23 days) F0 mice were randomly allocated into 3 groups: standard chow [control (CTRL)], high-fat diet (HFD) (#F3282, BioServ, USA—Carbohydrate: 35.7%, Protein: 20.5%, Fat: 36.0–59.0% Kcals), and HFD transition (HFD_t_) (60 days HFD, plus 140 days of standard chow). F0 mice were mated with chow-fed and lean females, after 120 days on diet. F1 and F2 male mice were assigned to the same experimental group as their progenitors, being fed standard chow. Mice were sacrificed on the 200th day by cervical dislocation. For this experiment, the 200-day period was selected since by the 60th day mice have already completed 2 spermatogenic cycles and can be considered sexually mature and this period is crucial for male reproductive maturation [26]. A schematic model representation can be found in the Fig. 1. Details regarding diet consumption and body weight can be found in previous work by Crisóstomo L. and colleagues. Briefly, higher body weight was found in F0 HFD and HFD_t_ mice, in comparison to the CTRL group. However, after diet reversion, the HFD_t_ group began to lose weight, thus the HFD presented the highest weight at the end of the experiment. Even though no significant differences were found in the F1 and F2 generations after weaning, the F1 HFD group was significantly heavier before this period [24]. After sacrifice, prostates were collected and immediately frozen in liquid nitrogen. Tissue samples were kept at -80º C, then lyophilized and kept on a desiccator until further use. The model is compliant with the ARRIVE guidelines and was approved and licensed by the Portuguese Veterinarian and Food Department (DGAV) (0421/000/000/2016).

### RNA extraction from prostate tissue

RNA extraction was performed using the NZYTech Total RNA isolation kit (NZYTech, Lisbon, Portugal), as indicated by the manufacturer. First, tissue (7.0 mg) was mixed with 350 µL of buffer NR and 3.5 µL of β-mercaptoethanol. The lysate was transferred to a NZYSpin Homogenization column placed in a 2 mL collection tube, and centrifuged for 1 min at 11,000 × *g*. The flow-through was preserved and transferred into a new 1.5 mL centrifuge tube. Then, 350 µL of 70% ethanol was added and mixed. After being transferred to a NYZSpin Binding column, the lysate was centrifuged at 11,000 × *g* for 30 s. The flow-through was discarded. 90 µL of Digestion Buffer and 10 µL of DNase I were mixed to prepare the digestion mix for each isolation. Of this mix, 95 µL was added into the center of the silica membrane column and incubated at room temperature for 15 min. Next, 200 µL of Buffer NWR1 was added and centrifuged for 1 min at 11,000 × *g*, and the flow-through was discarded. Then, 600 µL of the Buffer NWR2 was added and centrifuged at 11,000 × *g* for 1 min. The flow-through was discarded, and the wash was repeated with 250 µL of Buffer NWR2 and centrifuged at 11,000 × *g* for 2 × 1 min to dry the column membrane. The column was transferred to a clean 1.5 mL RNase-free microcentrifuge tube. After, 40 µL of RNase-free water was added directly to the column membrane and centrifuged at 11,000 × *g* for 1 min to elute the RNA. RNA concentrations and absorbance ratios (A260/A280 and A260/A230) were determined by Synergy H1 microplate reader with a Take3 plate (BioTeK, Santa Clara, USA). The RNA samples were stored at -80º C.

### Polymerase chain reaction (PCR)

To confirm the presence of the PCa biomarkers in the mice prostate tissue, as well as to validate our results with a positive control, and optimize the conditions for each set of primers, we performed PCR. Total RNA was reversely transcribed by M-MuLV Reverse Transcriptase (NYZTech, Lisbon, Portugal) according to the referenced protocol [27]. The cDNA samples were stored at -20º C. PCR assays were performed to identify the PCa biomarkers, AR and HOXB13, in the prostate tissue, following the established protocol [27]. The annealing temperatures were adjusted for each pair of primers (Table 1).

### Real-time polymerase chain reaction (qPCR)

qPCR Green Master Mix (2x) (NZYTech, Lisbon, Portugal) containing DNA polymerase enzyme, buffer, and SYBR green nucleic acid stain, was used. For the qPCR assays, each sample was mixed with 0.64 µL of primer forward (10 µM), 0.64 µL of primer reverse (10 µM), and 8.0 µL of Master Mix, with a final volume adjusted to 16 µL with RNase-free water. The cDNA quantity added to the mix was adjusted for each set of primers to ensure a reaction efficiency of 90–110%. To better fit optimal temperatures of enzyme activation, DNA denaturation, annealing, and dissociation, qPCR cycles conditions were adjusted. The following protocol was used: 2 min and 5 s at 95º C for polymerase activation and DNA denaturation, followed by 30 s at the annealing temperature of each set of primers. The primer sequences, annealing temperatures, and the number of cycles can be consulted in Table 1. The melting curve was determined by an additional step: 1 min at 95º C, 30 s at 55º C, and 30 s at 95º C. qPCR reactions were carried out in duplicate, and the optical density was assessed by a CFX Connect™ Real-Time PCR Detection System (Bio-Rad, California, USA).

**Table 1:** Primer sequences and PCR and qPCR conditions used to assess gene expression and mRNA abundance in mouse prostate tissue. *β*2-microglobulin was used as a housekeeping control

Gene	GenBank	Primer sequence (5’-3’)	Amplicon Length	Annealing Temperature	Cycles
mAR	NM_013476.4	Sense: GACTACTCTGCCTCCGAAGTG Anti-sense: AGTTCTCCATCCAAGGTCCCA	106 bp	58º C	33
mHOXB13	NM_008267.4	Sense: GGAGGGGGTCGGAATCTAGT Anti-sense: GTTGACAGTTGGCATCAGCG	81 bp	58º C	38
mFTO	NM_011936.2	Sense: GTGTCTCGCATCCTCATCGG Anti-sense: AGCCTCTGTGTACTTGACCG	111 bp	60º C	40
mTNF-α	NM_001278601.1	Sense: ACCCTCACACTCACAAACCAC Anti-sense: ACAAGGTACAACCCATCGGC	134 bp	60º C	37
mHOXB13 methylated		Sense: TTTTTTTAGGTTATAGTTAATTAGCGT Anti-sense: AAAAAATCCTAAACGTTTTAAATCG	127 bp	52º C	35
mHOXB13 unmethylated		Sense: TTTTTTAGGTTATAGTTAATTAGTGT Anti-sense: AAAAATCCTAAACATTTTAAATCACT	125 bp	52º C	35
mβ2-MGB		Sense: ACGTAACACAGTTCCACCCG Anti-sense: TCTCGATCCCAGTAGACGGT	217 bp	58º C (PCR) 60º C (qPCR)	35

### DNA extraction from prostate tissue

The NZYTech Tissue gDNA Isolation kit was used to perform DNA extraction, as indicated by the manufacturer. First, tissue (5.0 mg) was added to 180 µL of buffer NT1 and 25 µL of Proteinase K solution and mixed by vortex, followed by incubation at 56º C for 2 h, with occasional vortex. To each sample, 10 µL of RNase A solution was added and incubated for 5 min at room temperature. The samples were mixed by vortex, and 200 µL of Buffer NL was added. Then, 210 µL of 100% ethanol was added and mixed by vortex. The samples were transferred to an NYZSpin Tissue column, placed in a 2 mL collection tube, and centrifuged at 11,000 × *g* for 1 min. The flow-through was discarded and transferred into a new collection tube. Next, 500 µL of Buffer NW1 was added, being centrifuged for 1 min at 11,000 × *g*, and the flow-through was discarded. Then, 600 µL of the Buffer NW2 was added and centrifuged at 11,000 × *g* for 1 min, with the flow-through being discarded. The tissue column was transferred to a new collection tube and centrifuged for 2 min at 11,000 × *g*. The column was placed in a clean microcentrifuge tube, and added 100 µL of water, previously pre-heated at 70º C, to the membrane column. The samples were incubated for 1 min at room temperature and centrifuged at 11,000 × *g* for 2 min to elute DNA. DNA concentrations and absorbance ratios (A260/A280 and A260/A230) were determined by Synergy H1 microplate reader with a Take3 plate (BioTeK, Santa Clara, USA). The DNA samples were stored at -80º C.

### Evaluation of HOXB13 promoter methylation

Zymo Research EZ DNA Methylation™ Kit was used for total DNA conversion. First, CT Conversion Reagent was prepared by adding 750 µL of water and 210 µL of M-Dilution Buffer to a tube of CT Conversion Reagent, mixed by vortex at room temperature. To the total DNA samples (1 ng), 5 µL of M-Dilution Buffer was added and the total volume was adjusted to 50 µL with water and mixed by pipetting up and down. The samples were incubated at 37º C for 15 min. After, 100 µL of the CT Conversion Reagent was added and mixed. The samples were incubated in the dark at 50º C for 16 h and then in ice for 10 min. Meanwhile, 400 µL of M-Binding Buffer was added to a Zymo-Spin IC Column placed in a Collection tube. After incubation, samples were added to the column and mixed by inverting the column several times. The samples were centrifuged at 10,000 × *g* for 30 s and the flow-through was discarded. Then, 100 µL of M-Wash Buffer was added to the column and centrifuged at 10,000 × *g* for 30 s. To the samples was added 200 µL of M-Desulphonation Buffer and incubated for 15 min at room temperature, followed by centrifugation at 10,000 × *g* for 30 s. Then, 200 µL of M-Wash Buffer was added to the column and centrifuged at 10,000 × *g* for 30 s. This step was repeated another time. The column was replaced into a 1.5 mL microcentrifuge tube and added 10 µL of M-Elution Buffer directly into the matrix, being then centrifuged at 10,000 × *g* for 30 s. DNA-converted samples were stored at -20º C.

### Slot-Blot

Total proteins were extracted from lyophilized prostate tissue using RIPA buffer [1% Noridet P-40, 0.5% Sodium deoxycholate, 0.1% Sodium dodecyl sulfate 10% in phosphate buffer saline (PBS), 0.1% Phenylmethanesulfonyl fluoride 100 mM, 0.1% cocktail mix of protease and phosphatase inhibitors, 0.1% sodium orthovanadate 100 mM] and homogenized with microtubule piston. The samples were left to rest for 40 min at 4º C and then centrifuged at 14,000 × *g*, 4º C, 1 min. Protein concentration was determined by Pierce Bicinchoninic acid protein assay kit (Thermo Fisher Scientific, Massachusetts, USA). Lipid peroxidation and protein nitration were evaluated by Slot-Blot. Briefly, protein samples (5 µg) were diluted in PBS in a final volume of 100 µL. The samples were transferred to activated polyvinylidene difluoride membranes by a Hybri-slot manifold system (Biometra, Göttingen, Germany). For total protein normalization and loading control, a Ponceau S staining solution (Merck, Millipore, Massachusetts, USA) was used. The membranes were incubated with a blocking solution of washing buffer (Tris-buffered saline solution with 0.05% Tween 20) with 5% Bovine Serum Albumin (BSA) for 1 h. The membranes were washed 3 times for 5 min with washing buffer and incubated overnight (4º C) with the primary antibody diluted in washing buffer with 1% BSA. The list of antibodies and their concentrations are presented in Table 2. After, the membranes were washed with the washing buffer 3 times for 5 min and exposed to the secondary antibody diluted in washing buffer with 1% BSA for 1 h, at room temperature. Afterward, the membranes were washed and reacted with ethyl chloroformate fluorescent (ECF) substrate (GE Healthcare, Buckinghamshire, UK). Results were visualized by ChemiDoc^Tm^ MP Imaging system (Bio-Rad, California, USA). Densities from each band were quantified by BioRad ImageLab Software.

**Table 2:** List of antibodies, and respective concentrations, used for Slot-Blot

Antibody	Company	Catalog Number	Concentration for Slot-Blot
Rabbit Anti-nitro-tyrosine	Cell Signaling Technology	9691 S	1:1000
Rabbit Anti-4-hydroxynonenal	Abcam	Ab46545	1:1000
Anti-Rabbit IgC (whole molecule)– Alkaline Phosphatase antibody produced in goat	Sigma Aldrich	A3687–1 ML	1:5000

### Bioinformatic analysis

HOXB13 and Fat Mass and Obesity Associated gene (FTO) interactors experimentally identified in human and mouse were retrieved from the Human Integrated Protein-protein Interaction Reference (HIPPIE) and Mouse Integrated Protein-protein Interaction Reference (MIPPIE) public databases, respectively (consulted on Dec 11, 2023), using the respective Uniprot ID (HOXB13: Q92826; FTO: Q9C0B1). Moreover, the proteome of the human and mouse prostate was obtained from Human Protein Atlas and Mouse Gene Expression Database, respectively. To identify the HOXB13 and FTO interactors presented in prostate tissue, a Veen diagram analysis was conducted using Jvenn tool (available at: https://jvenn.toulouse.inrae.fr/app/index.html). The interactors of HOXB13 and FTO identified in the human prostate were used to construct a protein-protein interaction network using the Cystoscape software (version 3.8.2).

### Statistical analysis

Statistical analysis of the expression of the PCa biomarkers, *FTO*, Tumor Necrosis Factor-Alpha (*TNF-α*), methylation levels, and OS was performed using analysis of variance (ANOVA) followed by a Tukey post-hoc test for multiple comparisons. Population normality was evaluated by the Shapiro-Wilk test and Kolmogorov-Smirnov test. Correlations were evaluated by computing Pearson correlation coefficients (r) assuming Gaussian distribution with a confidence interval of 95%. Values of *P* < 0.05 were considered statistically significant. The statistical analysis was performed using GraphPad Prism 8 (GraphPad Software Inc., SanDiego, CA, USA).

## Results

### The offspring of males exposed to a lifelong HFD present an increased transcript expression of *HOXB13*, *FTO* and *TNF-α* in prostate tissue, which is reversed in the F1 of the HFD_t_ group

**Fig. 2:**
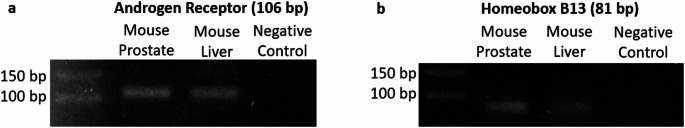
Identification of PCa biomarkers mRNA in mouse prostate tissue subjected to standard chow by conventional PCR. Conventional PCR was used to identify the presence of *Androgen Receptor* (**A**) and *Homeobox B13* (**B**) transcripts in mouse prostate tissue. A cDNA-free sample was used as a negative control. A mouse liver tissue sample was used as a positive control

**Fig. 3:**
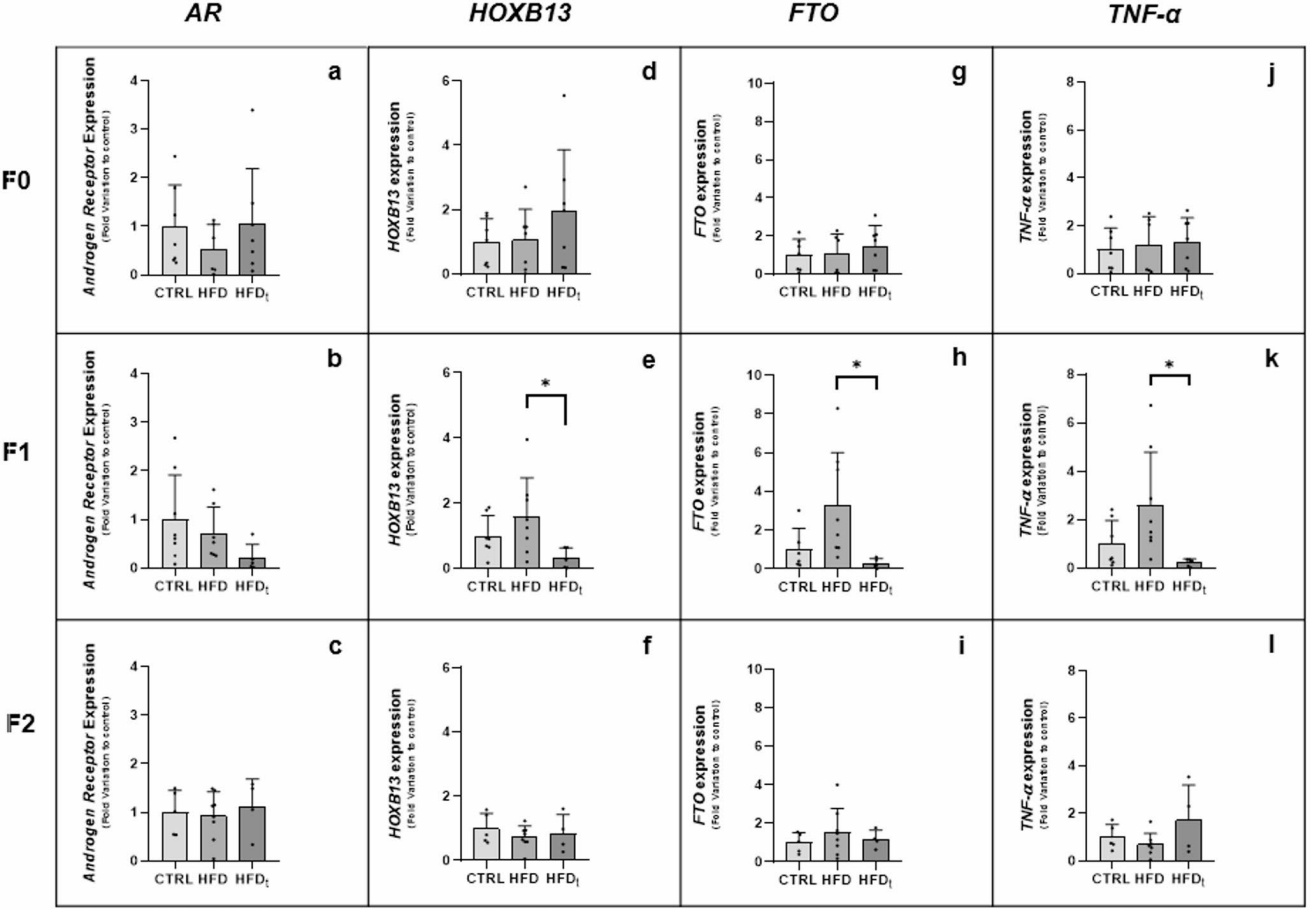
Expression of *AR* (**a**, **b**, **c**), *HOXB13* (**d**, **e**, **f**), *FTO* (**g**, **h**, **i**), and *TNF-α* (**j**, **k**, **l**) in the prostate of mice fed with standard chow (CTRL) or lifelong high-fat diet (HFD) or only early life HFD (HFD_t_) in F0 and on their offspring (F1 and F2). Data shows the expression of *AR*, *HOXB13*, *FTO*, and *TNF-α* in the prostate of mice (F0, F1, and F2). The expression was determined by qPCR. Results are expressed as mean ± SD, (*n* = 4–8 for each bar). Significantly different results (*p* < 0.05) are as indicated: * relative to HFD

We identified the expression of PCa biomarkers *AR*, and *HOXB13* in mice prostate tissue, through conventional PCR (Fig. 2). As expected, both were identified which is concomitant with previous reports [28, 29]. We hypothesized that distinct dietary regimens, particularly lifelong or early life obesity caused by HFD, could impact PCa biomarkers expression in the progenitors and in their offspring. Additionally, we focused on the potential effects of these dietary regimens on the expression of the obesity-related gene *FTO*, and the inflammatory biomarker *TNF-α* in the prostate tissue. AR is involved in the prostate’s development and functioning, while also influencing PCa progression. Here, *AR* prostate expression remained unaffected in all groups and generations (Fig. 3, Panel a, b, c). HOXB13 is related to PCa and obesity. *HOXB13* prostate expression presented a tendency towards an increase in the F0 HFD_t_ mice, relative to the CTRL group (Fig. 3, Panel d). However, their offspring (F1) presented a decrease in *HOXB13* prostate expression, compared to the F1 HFD group (Fig. 3, Panel e). FTO is a demethylase enzyme, implicated in the epigenetic modulation of the m6A (N6-methyladenosine) modification, which has the potential to influence *HOXB13* expression [30, 31]. In our study, *FTO* prostate expression presented a tendency towards an increase in the F0 HFD_t_ group (Fig. 3, Panel g), however, their offspring (F1) displayed a decrease, compared to the F1 HFD group (Fig. 3, Panel h). TNF-α plays a pivotal role in tumorigenesis, inclusively in PCa development. The F1 HFD_t_-fed mice presented a decrease in *TNF-α* prostate levels, compared to the F1 HFD-fed mice (Fig. 3, Panel k). The significant difference observed in the prostate of mice subjected to lifelong HFD or only during early life echoes a pattern reminiscent of the fluctuations observed in *HOXB13*, *FTO* and *TNF-α* expression.

### Positive correlation between the expression of *HOXB13* and *AR* in the prostates of progenitors and F1

We hypothesized a potential correlation between *HOXB13* and *AR* prostate expression of mice subjected to diverse dietary regimens across three generations. A positive correlation was found between *HOXB13* and *AR* prostate expression in F0 (Table 3, F0, *r* = 0.6068) and F1 (Table 3, F1, *r* = 0.7341). No correlation was found in F2 (data not shown). These results confirm a positive correlation according to the dietary regime (F0) that is maintained in the offspring (F1) and lost in the grand offspring (F2). Next, we identified a positive correlation between *FTO* and *HOXB13* prostate expression in F0 (Table 3, F0, *r* = 0.7714) and F1 (Table 3, F1, *r* = 0.7654), and between *FTO* and *AR* prostate expression in F0 (Table 3, F0, *r* = 0.9113) and F1 (Table 3, F1, *r* = 0.5905). Again, no correlation was found between the expression of *HOXB13* and *FTO* nor *AR* and *FTO* in F2 (data not shown), supporting that these prostate mediators respond to dietary regimens and their expression is influenced up to F2. Focusing on the link between obesity and inflammation, we analyzed a possible correlation between *TNF-α* and *FTO* prostate expression. *TNF-α* and *FTO* prostate expression were positively correlated in F0 (Table 3, F0, *r* = 0.9049) and F1 (Table 3, F1, *r* = 0.7027). Focusing on PCa biomarkers, we uncovered a positive correlation between *TNF-α* and *HOXB13* prostate expression in F0 (Table 3, F0, *r* = 0.6403) and F1 (Table 3, F1, *r* = 0.7580). No correlation was found between *HOXB13* and *FTO* with *TNF-α* in F2 (data not shown). Interestingly, *AR* was positively correlated with *TNF-α* prostate expression in F0 (Table 3, *r* = 0.8569).

**Table 3:** Correlation between the expression of *AR* and *HOXB13*, *FTO* and *HOXB13*, *FTO* and *AR*, *FTO* and *TNF-α*, *HOXB13* and *TNF-α*, *AR* and *TNF-α* in the prostate of mice fed with standard Chow (CTRL) or lifelong high-fat diet (HFD) or early life HFD (HFD_t_) in F0 and their offspring (F1). Table shows the correlation between *AR* and *HOXB13* expression in the prostate of F0 (*n* = 17) and F1 (*n* = 16) mice, between *FTO* and *HOXB13* expression in the prostate of F0 (*n* = 19) and F1 (*n* = 15) mice, between *FTO* and *AR* expression in the prostate of F0 (*n* = 18) and F1 (*n* = 16) mice, between *FTO* and *TNF-α* expression in the prostate of F0 (*n* = 20) and F1 (*n* = 16) mice, between *HOXB13* and *TNF-α* expression in the prostate of F0 (*n* = 19) and F1 (*n* = 16) mice, between *AR* and *TNF-α* expression in the prostate of F0 (*n* = 18) mice. Outliers were identified by ROUT (Q = 10%). All P values < 0.05 were considered statistically significant

		HOXB13	AR	FTO	TNF-α
*HOXB13*	F0		*r* = 0.6068 *p* = 0.0098	*r* = 0.7714 *p* = 0.0001	*r* = 0.6403 *p* = 0.0031
	F1		*r* = 0.7341 *p* = 0.0012	*r* = 0.7654 *p* = 0.0009	*r* = 0.7580 *p* = 0.0007
*AR*	F0			*r* = 0.9113 *p* < 0.0001	*r* = 0.8569 *p* < 0.0001
	F1			*r* = 0.5905 *p* = 0.0160	
*FTO*	F0				*r* = 0.9049 *p* < 0.0001
	F1				*r* = 0.7027 *p* = 0.0024
*TNF-α*	F0				
	F1				

### *HOXB13* promoter methylation levels are correlated with *HOXB13* expression in the prostate of progenitors but not in their offspring

**Fig. 4:**
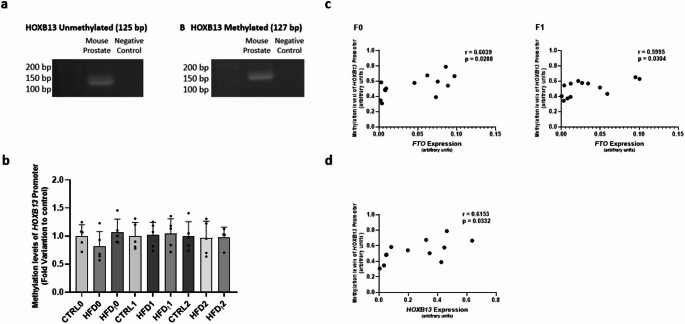
Identification of methylation-specific primers, *HOXB13* unmethylated and *HOXB13* methylated, in mice prostate tissue by MSP (**a**), methylation levels of *HOXB13* promoter (**b**) and correlation between *HOXB13* promoter methylation levels and *FTO* (**c**) and *HOXB13* (d) expression in the prostate of mice fed with standard chow (CTRL) or lifelong high-fat diet (HFD) or early life HFD (HFD_t_) in F0 and on their offspring (F1, and F2). Panel a shows the identification of the presence of unmethylated *HOXB13* and methylated *HOXB13* transcripts in mice prostate tissue through MSP. A cDNA-free sample was used as the negative control. Panel b shows the methylation levels of the *HOXB13* promoter in the prostate of mice. The expression was determined by qMSP. Results are expressed as mean ± SD (*n* = 5 for each graph bar), except for the group HFD_t_ in F2 (*n* = 4). Panel c shows the correlation between *HOXB13* promoter methylation levels and *FTO* expression in the prostate of F0 (*n* = 13) and F1 (*n* = 14) mice. Panel d shows the correlation between *HOXB13* promoter methylation levels and *HOXB13* expression in the prostate of F0 (*n* = 13) mice. Outliers were identified by ROUT (Q = 10%). All P values < 0.05 were considered statistically significant

We further investigated the HOXB13 promoter methylation patterns to determine their potential connection with *HOXB13* expression, subsequently extending to the relationship with *FTO* expression. Methylation-specific PCR (MSP) was used to validate *HOXB13* promoter transcripts existence in mice prostate tissue (Fig. 4, Panel a) and to ensure the accurate conversion of all samples, followed by Quantitative MSP (qMSP) to gauge HOXB13 promoter methylation levels. The outcomes of methylation analysis were depicted as a ratio between methylated and unmethylated levels. Intriguingly, results revealed no alterations in the *HOXB13* promoter methylation levels (Fig. 4, Panel b). The overall implication suggests that fluctuations in *HOXB13* prostate expression may not be intricately linked to variations in promoter methylation levels. Considering prior research, we aimed to explore a potential correlation between *HOXB13* promoter methylation levels and *HOXB13* and *FTO* prostate expression. Our findings revealed a positive correlation between *FTO* expression and *HOXB13* promoter methylation levels in F0 (Fig. 4, Panel c, F0, *r* = 0.6039) and F1 (Fig. 4, Panel c, F1, *r* = 0.5995), which was not observed in F2 (data not shown). Furthermore, *HOXB13* expression was positively correlated with *HOXB13* promoter methylation levels in F0 (Fig. 4, Panel d, *r* = 0.6153).

### Lipid peroxidation levels are increased in the prostate of mice fed lifelong HFD but not in the prostate of their offspring

**Fig. 5:**
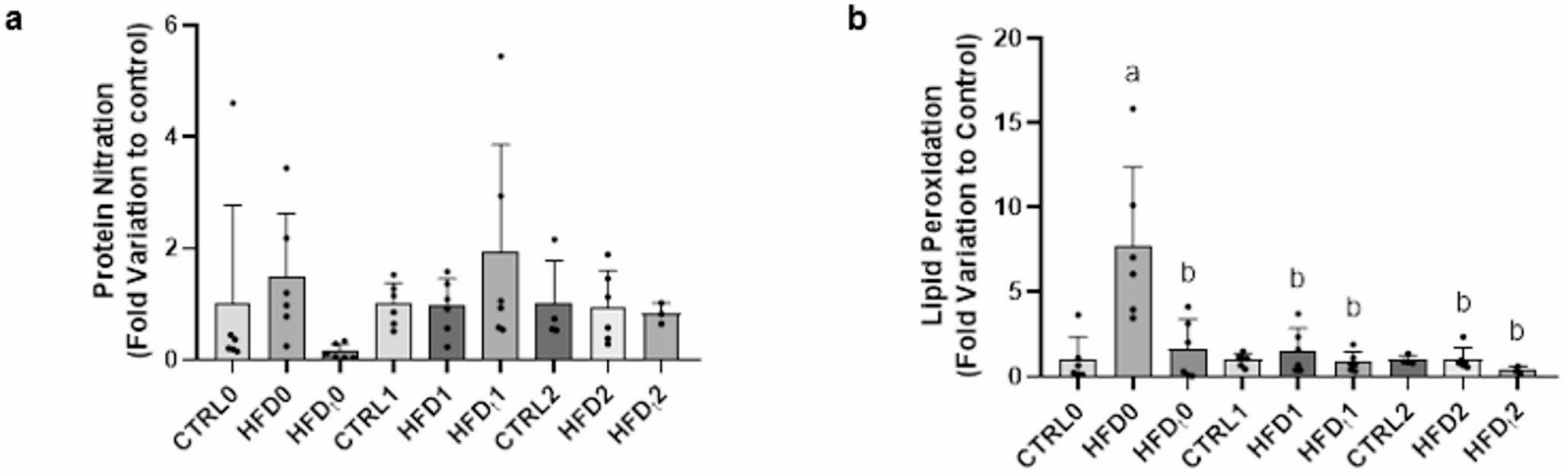
Evaluation of protein nitration (**a**) and lipid peroxidation (**b**) in the prostate of mice fed with standard chow (CTRL) or lifelong high-fat diet (HFD) or early life HFD (HFD_t_) of progenitors in F0 and on their offspring (F1, and F2). Data shows the analysis of protein nitration (**a**) and lipid peroxidation (**b**) in the prostate of mice of F0, F1, and F2. The expression was determined by Slot-Blot. Results are expressed as mean ± SD (*n* = 6 for each graph bar), except for the groups CTRL (*n* = 4) and HFD_t_ (*n* = 3) in F2. Significantly different results (*p* < 0.05) are as indicated: a relative to CTRL, b relative to HFD

We proposed to study OS-related deleterious markers in the prostate of mice subjected to different dietary regimens and on their offspring. Protein nitration results in the prostate were unaffected in all groups and generations (Fig. 5, Panel a). Regarding lipid peroxidation, the F0 HFD group exhibited an increase, when compared to the CTRL and HFD_t_ groups. In F1 and F2, the lipid peroxidation levels of the HFD and HFD_t_-fed mice progeny were restored to normal values when compared to F0 HFD-fed mice (Fig. 5, Panel b). Representative and uncropped membranes of these essays can be found as supplementary information.

### No common proteins have been described between the interactome of FTO and HOXB13 in the human and mouse prostate

**Fig. 6:**
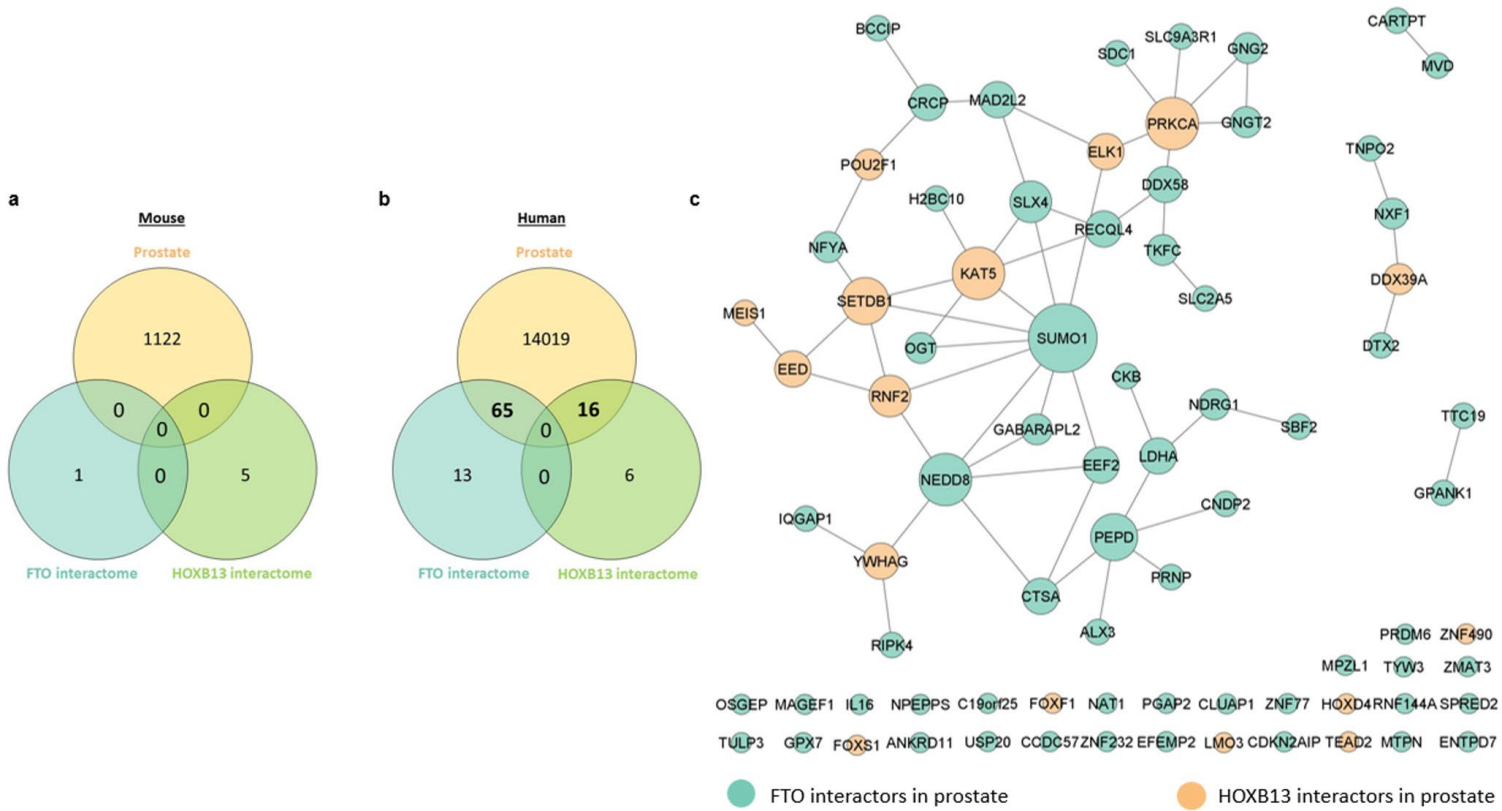
Venn diagram illustrating the proteins present in mouse (**a**) and human (**b**) prostate that interact with FTO and/or HOXB13 and protein-protein interactions network of the identified FTO and HOXB13 interactors in human prostate (**c**). The node size is proportional to the number of interactions

To unveil the relation between FTO and HOXB13 in the prostate, we performed a bioinformatic analysis focused on the study of potential interactions (Fig. 6, Panel a and b). In mice prostate, no FTO or HOXB13 interactors were identified (Fig. 6, Panel a). In human prostate, no discernable common interactors between FTO and HOXB13 are presented, however, FTO and HOXB13 interact with several proteins already identified, with FTO exhibiting a higher number of interactions (65) compared to HOXB13 (16) (Fig. 6, Panel b). Notably, the lack of documented interactions between FTO and HOXB13 in the prostate or any other organ, in mice and humans, does not conclusively negate their existence. Considering the preliminary nature of the investigation into the association of FTO and HOXB13 in the prostate, further research in this area is warranted. The subsequent bioinformatic analysis focused on the interplay among FTO and HOXB13 interactors in the human prostate (Fig. 6, Panel c). Noteworthy, FTO exhibits a higher number of interactor partners in the prostate compared to HOXB13. Among these, Small Ubiquitin-related Modifier 1 (SUMO1), Lysine acetyltransferase 5 (KAT5), Protein Kinase C alpha (PRKCA), and SET Domain-bifurcated Histone Lysine Methyltransferase 1 (SETDB1), emerge as particularly relevant interactors. SUMO1, a FTO interactor, is directly linked to KAT5 and SETDB1, which are HOXB13 interactors. Conversely, KAT5 and SETDB1 demonstrate a correlation between them. Although the HOXB13 interactor, PRKCA, is not directly associated with any of the aforementioned interactors, it is indirectly connected to SUMO1.

## Discussion

In this study, we aimed to contribute to the gap of whether there is a correlation between the inheritance of obesity-related factors and their contribution to PCa development by identifying the response of PCa biomarkers to distinct dietary regimens, lifelong HFD or HFD only during early life followed by normal diet. Additionally, we aimed to determine whether dietary modifications could lead to alterations transmitted to subsequent generations, thereby increasing susceptibility to PCa development.

AR is crucial for the development and correct function of the prostate [13]. In our model, *AR* prostate levels were unaffected by dietary regimens. Previous findings reported that an HFD consumption can affect AR expression, potentially fostering carcinogenesis [32]. Thus, considering the mice body weight data previously mentioned, specifically the significant results in the F0 generation, we hypothesize that the percentage of fat and duration of diet consumption may be factors to consider regarding a potential impact on *AR* expression. Conversely, HOXB13 plays a pivotal role in the proper development of the prostate [15]. Concurrently, *de novo* lipogenesis is inhibited as HOXB13 directly suppresses lipogenic transcriptional programs. Reduced *HOXB13* expression correlates with significant lipid buildup within PCa cells, increasing metastasis [33]. Here, *HOXB13* prostate expression decreased in the F1 HFD_t_ group, compared to the HFD group. Regarding the mice weight results, no differences are found for *HOXB13* in F0, hence the dietary regimens appear to not have an impact on *HOXB13* expression. Nonetheless, it is worth noting, in the F1 HFD group, that a higher *HOXB13* expression is followed by a higher body weight at weaning, indicating a potential switch on this network. Our findings suggest that even if male progenitors switch from an HFD to a standard diet after early life, their offspring may still face an increased expression of PCa biomarkers. This implies that diet correction may not adequately counteract the harmful impacts of HFD consumption on prostate health. We further expanded our studies to HOXB13, since it regulates AR, through the transcription repression or activation of various AR target genes [15]. A positive correlation between higher HOXB13 expression and heightened AR levels was found in PCa tissue samples, with HOXB13 overexpression being present in the majority of PCa cases, as well as malignant modification. Further, this correlation was found along with a downregulation of PSA levels, suggesting an important need for the inclusion of other biomarkers in the PCa diagnosis, given the possible occurrence of a faulty diagnosis [16]. Here, the PCa biomarkers were positively correlated in F0 and F1, but not in F2. These findings reinforce the existence of a link between *AR* and *HOXB13* expression, which may be associated with higher PCa risk. In addition, suggests that the impact of HFD consumption in these PCa biomarkers appears to be transmitted to the sons but not to the grandsons. Further research will be needed to confirm these findings.

To correlate obesity with PCa development, we analyzed the expression of the obesity-related gene, *FTO*, which is intricately linked with metabolic disorders and cancer [34]. We found a decrease in *FTO* prostate expression in the F1 HFD_t_ mice, compared to the HFD group. Despite the link between *FTO* and body weight, no significant differences are found in its expression when the body weight of mice are heightened in the F0 generation. Conversely, the increased *FTO* expression occurs in the F1 HFD group. Considering FTO’s association with obesity and PCa, we hypothesized a potential correlation between *FTO* and PCa biomarkers expression. Notably, FTO’s activity is linked with *HOXB13* expression through the removal of the m6A modification, with heightened *FTO* expression along with increased *HOXB13* expression being present in the progression of endometrial and gastric cancer [30, 31], yet no link has been described in PCa. Through m6A modification, AR may be related to FTO, that participates in modulating the proliferative capacity of undifferentiated spermatogonia and Leydig cells’ maturation in mice through AR regulation, dependent upon m6A modification [35]. Moreover, a significant association between AR and FTO levels has been described in metastatic PCa, whereas a reverse link was observed in primary PCa [36]. Likewise, a positive correlation was found in PCa between AR and FTO [37]. Our results uncovered a correlation between *FTO* and both *HOXB13* and *AR* prostate expression in F0 and F1. Intriguingly, this correlation diminishes in F2, indicating a tissue remodeling possibly associated with male progenitor dietary habits or transmission mechanisms. Further studies are needed to clarify the impact and interaction of *HOXB13* and *FTO* on PCa development. In addition, these results strengthen the link between *AR* and *FTO*, which could be implicated in PCa progression. To elucidate the underlying mechanisms governing the observed fluctuations in *HOXB13* prostate expression, we assessed *HOXB13* promoter methylation levels. Promoter hypermethylation leads to suppressed gene expression, a phenomenon linked to various cancers, including PCa [38]. Furthermore, it has been hypothesized that *FTO* might decrease *HOXB13* promoter methylation levels, enhancing *HOXB13* expression, which was linked to gastric cancer cell proliferation and invasion [30]. Our findings indicate an absence of differences in the *HOXB13* promoter methylation levels, suggesting that the gene expression is not directly influenced by alterations in promoter methylation levels. Further, we explored the possibility of a correlation between *HOXB13* promoter methylation levels, *FTO* and *HOXB13* expression. *FTO* expression was positively correlated with *HOXB13* promoter methylation levels in F0 and F1. Interestingly, in gastric cancer, *FTO* was proposed to decrease *HOXB13* methylation [30]. Moreover, *HOXB13* promoter methylation levels and *HOXB13* expression were positively correlated in F0. Recent evidence has presented that hypermethylation is linked to increased gene expression, with certain genes having complex methylation signaling [39], thus explaining the different pattern identified in our model.

Obesity promotes an inflammatory state that may also be associated with carcinogenesis and PCa. Thus, we studied the expression of *TNF-α*, an inflammatory biomarker. *TNF-α* prostate expression was elevated in F1 HFD mice, compared to the HFD_t_ group. Previous findings reported increased TNF-α expression linked to PCa [40] and to an inflammatory state due to HFD consumption [41]. Regardless, no significant differences were found in its expression when mice body weight was augmented in the F0 generation. However, increased *TNF-α* expression was obtained in the F1 HFD group. Given the striking similarity between *TNF-α* expression patterns to those observed in *HOXB13* and *FTO* expression, we evaluated potential correlations among all markers. Interestingly, we identified a positive correlation between *TNF-α* and *FTO* prostate expression in F0 and F1. These findings indicate that the correlation between obesity and prostate inflammation is influenced by dietary patterns and may impact future generations. For instance, a correlation was established between *TNF-α* and *FTO* expression in subcutaneous adipose tissue [42]. Further, *TNF-α* and *HOXB13* expression were positively correlated in F0 and F1. As of now, there is no established direct correlation between TNF-α and HOXB13 and the underlying mechanism governing their association remains to be elucidated in PCa. Nonetheless, a study with transgenic mice skin observed an increase in TNF-α levels concomitantly with HOXB13 overexpression. Authors suggested that HOXB13 could promote the expression of TNF-α through its role as a transcription factor or indirectly by regulating upstream genes, providing a possibility for their regulatory link [43]. Additionally, *AR* expression presented a positive correlation with *TNF-α* expression in F0. Conversely, in human prostate cells, TNF-α activates the Nuclear Factor Kappa B (NF-kB), inhibiting *AR* expression, that consequently decreased androgen sensitivity in androgen-dependent LNCaP cells [44]. Thus, our findings further suggest that dietary regimens, particularly HFD consumption, could impact this network, substantiating an interplay which displays a possible impact on PCa dynamics. Further studies will be needed to unveil the consequences of the disruption of this pathway for prostate health.

We performed a bioinformatic analysis and detected no interactions between HOXB13 and FTO in the prostate of mouse or human models. Noteworthy, these results do not imply the non-existence of interactions, which most possibly have not been reported yet. Still, multiple connections between FTO and HOXB13 interactors, namely KAT5, PRKCA, SETDB1, and SUMO1 were identified, which have been linked to carcinogenesis and PCa development. SETDB1 can exert its influence in carcinogenesis and metastasis [45]. In PCa, a heightened SETDB1 expression is found, while its silencing resulted in inhibited cell proliferation and invasion [46]. PRKCA participates in processes intricately linked to tumor-promoting events [47], and in PC-3 and DU-145 cell lines exhibits upregulation [48], whereas, in LNCaP cells, the apoptotic response induced by Phorbol 12-myristate 13-acetate (PMA) is promoted by NF-kB and active PRKCA mutants [49]. Concurrently, PRKCA activation can result in AKT inactivation, an event associated with apoptosis in PCa cells [49]. The sumoylation process, involving SUMO1, intervenes in the regulation of cancer-related pathways, inclusively NF-kB [50]. A mechanism that might influence PCa development is the Phosphatidylinositol 3-kinase–AKT (PI3K-AKT) pathway, which can be affected by the SUMO1 modification of PTEN [51]. KAT5 is a lysine acetyltransferase that acetylates several targets, including NF-kB [52], with increased expression described in aggressive PCa and as a Castration-resistant Prostate Cancer (CRPC) initiator [53]. Notably, in DU-145 cells, KAT5 and KAT6B silencing inhibited PI3K signaling, along with downstream AKT and NF-kB signaling pathways. The AKT signaling suppression underscores the role of these enzymes in regulating PCa growth through the PI3K-AKT pathway [53].

A common point has been the link of PCa biomarkers, obesity-related factors, as well as KAT5, PRKCA, and SUMO1 to NF-kB, a transcription factor, involved in proliferation, inflammation, and apoptosis, all tumor-promoting processes. Curiously, TNF-α was correlated with NF-kB, and this association was linked to tumor size and stage in breast cancer tissue [54]. Further, HOXB13 participates in the NF-kB pathway, contributing to PCa progression [55]. Concerning FTO, to our knowledge no association has been yet established with NF-kB in PCa. Given our data, alterations in the NF-kB pathway may be the link between obesity and PCa. We hypothesize that this pathway could also play a role in the hereditary transmission of PCa. It is important to acknowledge some limitations concerning this study. First, we have a limited size of samples, which only allowed us to perform the tests mentioned in the methods section. A histopathological analysis would be interesting to perform to assess if mice prostates were carcinogenic or not. Unfortunately, the small size of the mouse prostate did not allow us to perform such analysis. Instead, we focus our work on the evaluation of PCa biomarkers expression and how a HFD could promote the alteration of their expression, as well as with the obesity-related markers. We must also highlight that the timeframe of this work did not allow us to evaluate the prolonged effects of a HFD exposure on mice’s prostate tissue.

## Conclusion

In conclusion, our work highlights the increased PCa risk in male mice fed an HFD throughout their lives or only during early life and underscores the higher PCa susceptibility among their offspring. Our findings unravel the intergenerational dynamics of dietary influence on metabolic and molecular alterations, resulting in a heightened PCa risk development. Further, these alterations appear to be resistant to diet correction in exposed and unexposed generations, though they tend to disappear in the F2 generation. Finally, this study highlights the complex interplay between obesity and PCa risk, emphasizing the link between obesity-related effects, inflammation and OS. Furthermore, it underscores the significance of paternal lifestyle choices on the health of subsequent generations, illustrating the far-reaching consequences of dietary habits, especially concerning PCa risk.

## Electronic supplementary material

Below is the link to the electronic supplementary material.

Supplementary Material 1

## Data Availability

Data will be made available on reasonable request.
