# Supplementary material for: Increased susceptibility to prostate cancer biomarkers in the offspring of male mouse progenitors with lifelong or early life exposure to high-fat diet
Source: Eur J Nutr. 2025 Jun 7;64(5):212. doi: 10.1007/s00394-025-03737-3 (PMC12145303; doi:10.1007/s00394-025-03737-3)
Supplement: Supplementary file 1 — Supplementary Material 1 [file 394_2025_3737_MOESM1_ESM.docx]

**Supplementary Information**


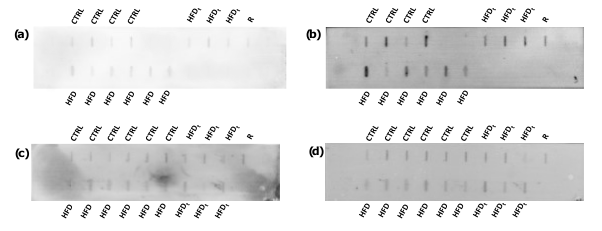


**Figure**. S1 Representative and uncropped image of a Slot-Blot membrane, regarding lipid peroxidation (a) and respective ponceau essay (b), as well as protein nitration (c) and respective ponceau essay (d), in the prostate of offspring of mice fed with standard chow (CTRL) or lifelong high-fat diet (HFD) or only until adulthood (HFD_t_), and a reference mouse prostate tissue sample (R).
